# Epidemiology and prognostic factors of Hürthle-oncocytic cell carcinoma of the thyroid

**DOI:** 10.1007/s12672-026-04442-1

**Published:** 2026-02-02

**Authors:** Omar Hamdy, Hedaa Atwa, Ekbal Elkhouli, Ahmed H. Ata, Radwa M. Abdelsattar, Maryam Dawood, Shadi Awny, Mohamed Ezat

**Affiliations:** 1 Surgical Oncology Department, Oncology Center, Mansoura University, Mansoura, Egypt; 2 Pathology Department, Faculty of Medicine, Mansoura University, Mansoura, Egypt; 3 Mansoura University Hospitals, Mansoura, Egypt

**Keywords:** Hürthle cell carcinoma, Thyroid cancer, Survival, Recurrence, Oncocytic carcinoma

## Abstract

**Introduction:**

Hürthle cell carcinoma (HCC) -recently known as oncocytic carcinoma- is a rare type of differentiated thyroid cancer that presents a diagnostic and therapeutic challenge because of its morphological heterogeneity and uncertain biological behavior.

**Methods:**

This retrospective single-center cohort study included all the patients with HCC who underwent surgical treatment in our center from January 2009 to May 2024. The epidemiological, clinical, and oncological data of the included patients were analyzed.

**Results:**

This study included nineteen cases of HCC (9 males and 10 females). The average age at diagnosis was 54.8 ± 12.2 years. Preoperative fine needle aspiration cytology (FNAC) classified 2 tumors as Bethesda I, 7 as Bethesda III, 6 as Bethesda IV, and 4 as Bethesda V. A variety of surgical procedures were used, including hemithyroidectomy in 3 patients and total thyroidectomy in 12 patients. Two patients underwent neck dissection. The median tumor size was 6.7 cm. Pathological evaluation identified 9 patients with unifocal lesions and 10 with multifocal lesions. Only one patient showed positive lymph node involvement. The median times to death, distant metastasis, and locoregional recurrence were 4, 13, and 6 years, respectively. For locoregional recurrence, the restricted mean survival time (RMST) at five years was 4.4 years (95% CI 3.9–4.9), 4.6 years (95% CI 4.1–5.0) for distant metastasis, and 4.1 years (95% CI 3.6–4.5) for overall survival. There was a trend towards worse prognosis in females, younger age, and those with primary surgery outside the center. These differences did not achieve statistical significance, at least partly due to the small sample size.

**Conclusion:**

Diagnosing HCC remains challenging due to its overlapping features with other thyroid conditions, making fine-needle aspiration cytology less definitive. Surgical treatment remains the preferred therapeutic option. Age, gender, and the volume of the surgical center for the initial procedure can influence patient outcomes, particularly recurrence and survival rates.

## Introduction

Hürthle cell carcinoma (HCC), also termed oncocytic carcinoma in the recent WHO classification [1–3]- is a rare and aggressive variant of differentiated thyroid cancer that accounts for approximately 3–5% of all thyroid malignancies and is defined by the presence of at least 75% Hürthle -oncocytic- cells [4–6]. HCC is relatively more common in women and presents as painless cervical nodules, often associated with multinodular goiter [4].

The clinical course of these cancers ranges from indolent to aggressive [7], creating diagnostic and therapeutic challenges because of their morphological heterogeneity and uncertain biological behaviour. Distniguishing Hürthle cell adenoma from carcinoma is difficult, as the diagnosis relies on demonstrating capsular and/or vascular invasion, which can be challenging to assess and variably interpreted [6]. Although a reliable preoperative diagnosis of these tumors is vital for management, fine-needle aspiration cytology (FNAC) - which is the primary diagnostic modality – has limitations in providing a definite diagnosis due to overlapping cytological features with other thyroid lesions [8–10].

Management of HCC remains controversial, particularly regarding optimal extent of surgery, the benefit of adjuvant therapies, and the long-term outcomes related to recurrence and survival [11]. Following surgery, radioactive iodine (RAI) ablation may be considered. Also, it has exhibited better survival rates in RAI-concentrating tumors in metastatic disease. Tyrosine kinase inhibitors represent a cornerstone of treatment in RAI-refractory thyroid cancer, particularly in metastatic and symptomatic cases [12].

The study aims to characterize this rare thyroid malignancy by presenting a 15-year experience from a tertiary cancer center, focusing on its presentation, diagnosis, management, and outcomes.

## Methods

This retrospective study included all patients with Hürthle cell carcinoma of the thyroid gland who underwent surgical treatment in our center from January 2009 to May 2024. Institutional Research Board (IRB) approval was received for the study from the medical research ethics committee at Mansoura University Faculty of Medicine under the number R.24.07.2692.

### Inclusion criteria

Patients with pathologically proven HCC who underwent surgical management during the study period.

### Exclusion criteria

• Missing or unregistered data.
• No surgical management.
• Unconfirmed diagnosis.

The following data were collected for every patient:

• Epidemiological: age, gender, medical comorbidities.
• Clinical: complaint, site of the nodule, size, focality, laterality, lymphadenopathy, Thyroid Imaging, Reporting and Data System (TIRADS), FNAC, and type of surgery.
• Oncological: pathological type, size, lymph node involvement, stage, adjuvant treatment, recurrence, metastasis, and survival.

### Primary outcome

Disease-free survival.

### Secondary outcome

Recurrence, distant metastases, and overall survival.

### Pathology review

An independent pathologist reviewed the pathology slides of the included patients in view of the recent WHO classification (2022). Those with a different diagnosis were excluded from the study.

### Statistical analysis

Data were entered and analyzed using IBM SPSS software (IBM Corp. Released 2020. IBM SPSS Statistics for Windows, Version 27.0. Armonk, NY: IBM Corp. Also, MedCalc software (version 18.9.1) was used for restricted mean survival time (RMST) analysis. Qualitative data were expressed as frequency (N) and percentage (%). Quantitative data were initially tested for normality using Shapiro-Wilk’s test, with data being normally distributed if *p* > 0.05. The presence of significant outliers was tested by inspecting the boxplots. Quantitative data were expressed as mean ± standard deviation (SD), or median and Q1-Q3 (25th – 75th percentiles). For survival analysis, the Kaplan-Meier method was used to estimate the probability of survival past given time points (i.e., it calculates a survival distribution). The survival distributions of two or more groups of a between-subjects factor were compared for equality using the log-rank test. Cox proportional hazards regression was used to investigate the effect of a variable on the time a specified event takes to happen. The hazard ratio associated with a predictor variable is given by the exponent of its coefficient, with a 95% confidence interval. The RMST was reported with its 95% confidence interval at a 12-month time point. The 5-year time point for RMST was selected to balance the need for a clinically meaningful follow-up period with the limited number of events. Differences in RMST between groups were reported as p-values. For any of the tests used, results were considered statistically significant if the p-value *≤ 0.05*. Appropriate charts were used to graphically present the results whenever needed. Due to the small sample size, the study may be underpowered to detect statistically significant differences in prognostic factors. Effect sizes were considered to provide context for non-significant results.

## Results

This study included nineteen patients with HCC (9 males and 10 females). The average age at diagnosis was 54.8 ± 12.2 years. Fifteen patients presented to the center at the time of initial diagnosis, while four patients presented with recurrent lesions. The clinical and demographic data are summarized in Table 1.

**Table 1:** Clinical and demographic data

Variable	*N*	%
Sex		
Male	9	47.4
Female	10	52.6
Age (years) at diagnosis		
< 55	10	52.6
≥ 55	9	47.4
Comorbidity	7	36.8
Diabetes	5	26.3
Hypertension	4	21.1
Presentation		
Primary	15	78.9
Recurrent	4	21.1
Imaging		
Focality		
Unifocal	11	57.9
Multifocal	8	42.1
Laterality		
Unilateral	9	47.4
Bilateral	6	31.6
N/A	4	21.1
Suspicious lymph nodes		
Yes	2	10.5
No	13	68.4
N/A	4	21.1
TIRADS		
3	3	15.8
4	10	52.6
5	6	31.6
Preoperative FNAC (Bethesda)		
I	2	10.5
II	0	0
III	7	36.8
IV	6	31.6
V	4	21.1
Pathological variables		
Focality		
Unifocal	9	47.4
Multifocal	10	52.6
Laterality		
Unilateral	15	78.9
Bilateral	4	21.1
Lymphovascular emboli		
Yes	5	26.3
No	10	52.6
N/A	4	21.1
T stage		
1	2	10.5
2	8	42.1
3	9	47.4
N stage		
0	18	94.7
1	1	5.3
AJCC stage		
I	12	63.2
II	5	26.3
III	2	10.5
Numerical characteristic	Mean	SD
Age (years)	54.8	12.2
	Median	Q1-Q3
Hospital-stay (days)	2	2–3
Maximum diameter (cm)	4.3	3.4–6.4
Operative time (minutes)	108.8	90–150
Pathological size (cm)	6.7	4–8

*SD* standard deviation. Q1-Q3 = 25th – 75th percentiles

**Fig. 1:**
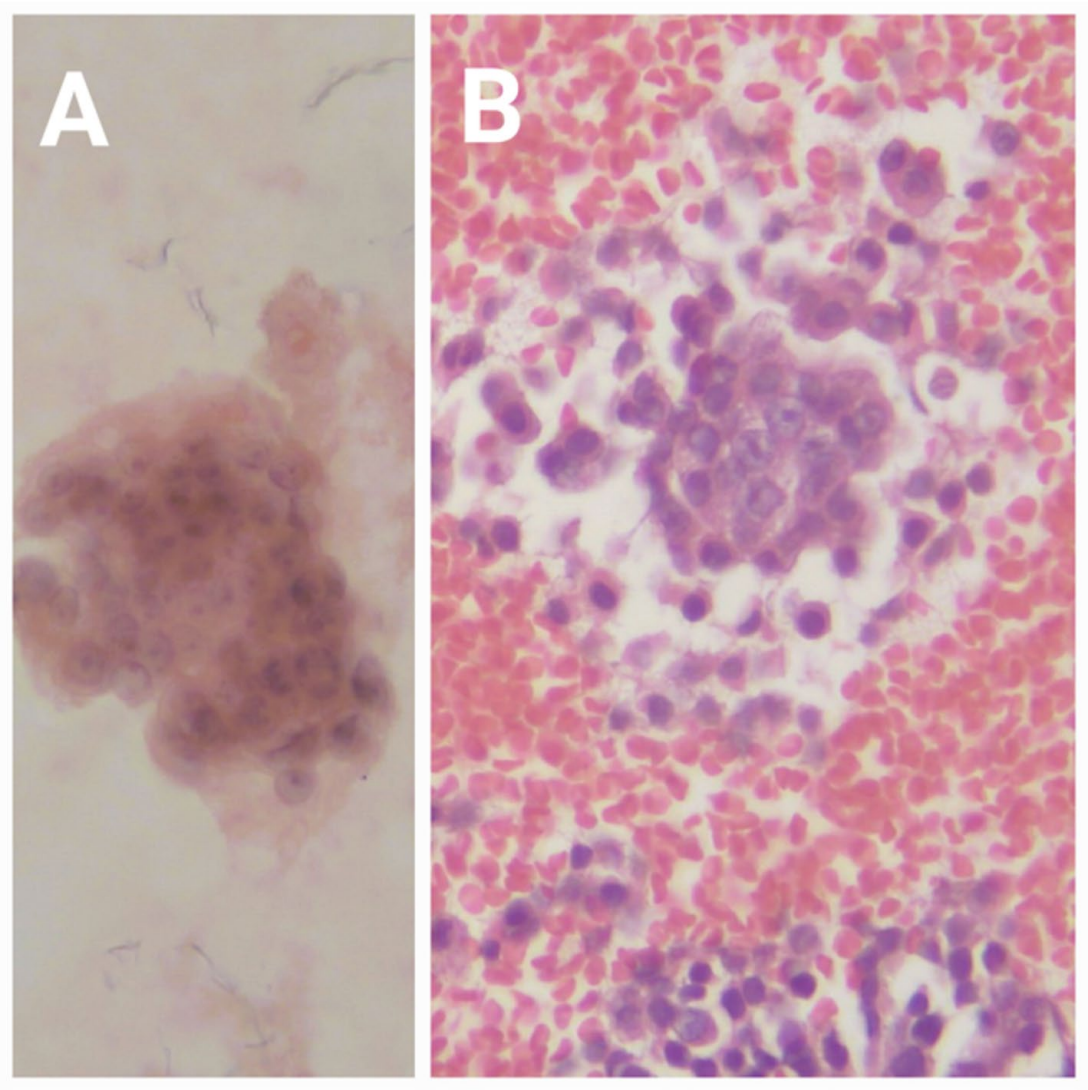
Microscopic examination of FNAC of HCC. **A** Fine needle aspiration cytology of another case of Hürthle cell carcinoma of the thyroid showing an aggregate of follicular cells forming microfollicles. The component cells show vesicular nuclei, overlapping each other, delicate, regular nuclear membranes, and prominent nucleoli. Cytoplasm is abundant and eosinophilic. Hematoxylin and Eosin stain, X200. **B** Cell block prepared from fine needle aspiration cytology of another case of Hürthle cell carcinoma of the thyroid, showing an aggregate of follicular cells forming microfollicles. The component cells show vesicular nuclei, overlapping each other, delicate, regular nuclear membranes, and prominent nucleoli. Cytoplasm is abundant and eosinophilic. Hematoxylin and Eosin stain, X200

On neck ultrasound, multifocal lesions were detected in 8 patients and unifocal lesions in 11 patients. The lesions were present in the right lobe, left lobe, and both lobes in 3,10, and 6 patients, respectively. Only two patients showed radiologically detected suspicious LNs (lymph nodes). The tumors were categorized as Thyroid Imaging Reporting and Data System TIRADS 3 (three patients), TIRADS 4 (ten patients), and TIRADS 5 (six patients). Preoperative FNAC classified two tumors as Bethesda I, seven as Bethesda III, six as Bethesda IV, and four as Bethesda V (Fig. 1).

A variety of surgical procedures were performed for the 15 patients who presented initially, including hemithyroidectomy in 3 patients and total thyroidectomy in 12 patients (whether on a single stage or as a completion thyroidectomy). Two patients underwent neck dissection. The median operative time was 120 min (range: 90–180 min), and the median hospital stay was 2 days (IQR:2–3 days).

**Fig. 2:**
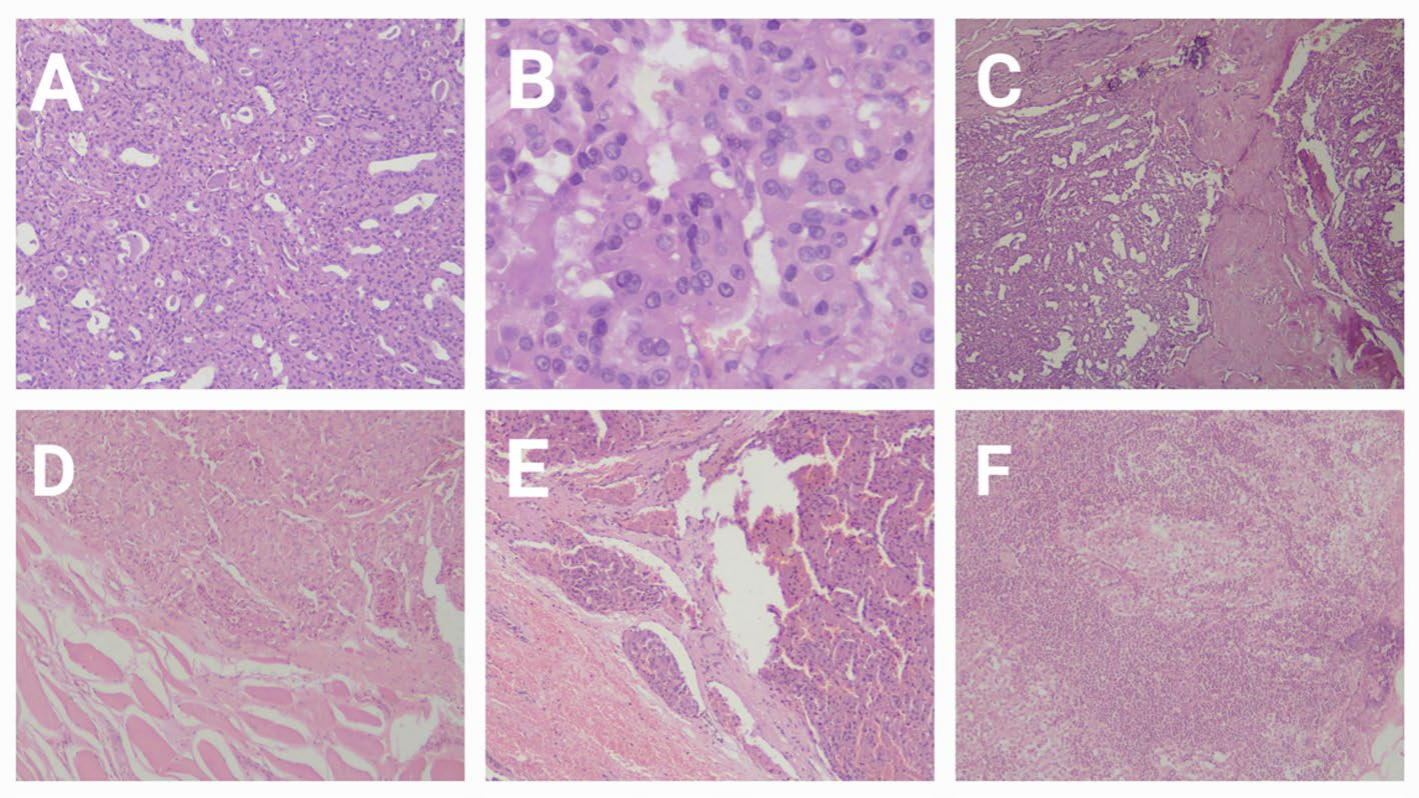
Microscopic examination of a surgical specimen of HCC. **A** A case of Hürthle cell carcinoma of the thyroid showing tumoral proliferation that is arranged in macro and microfollicular structures, together with solid areas. These are lined by bland-looking follicular cells that exhibit ample eosinophilic cytoplasm (Hürthle cells), imparting an eosinophilic low-power appearance to the lesion. Hematoxylin and Eosin stain, X40. **B** High power magnification of the same case of Hürthle cell carcinoma of the thyroid showing microfollicles which are anastomosing and formed of Hürthle cells that show ample eosinophilic granular cytoplasm, vesicular nuclei, and prominent nucleoli. Hematoxylin and Eosin-stain, X400. **C** The same case of Hürthle cell carcinoma of the thyroid showing an area of trans- capsular invasion in a mushroom-shaped fashion, Hematoxylin and Eosin-stain, X40. **D** The same case of Hürthle cell carcinoma of the thyroid showing an area of skeletal muscle fiber invasion at the advancing edge of the tumor, Hematoxylin and Eosin-stain, X400. **E** The same case of Hürthle cell carcinoma of the thyroid showing two vessels with angio-lymphatic tumor invasion at the advancing edge of the tumor, Hematoxylin and Eosin-stain, X100. **F** The same case of Hürthle cell carcinoma of the thyroid showing metastatic tumor deposits in the subcapsular sinus of a regional lymph node and replacing lymph nodal tissue, Hematoxylin and Eosin-stain, X40

The median tumor size, as determined by postoperative histopathological assessment of Hematoxylin & Eosin-stained slides (Fig. 2), was 6.7 cm (IQR: 4–8 cm). Pathological evaluation identified 9 patients with unifocal lesions and 10 with multifocal lesions (6 unilateral and 4 bilateral). It also revealed lymphovascular emboli in 6 patients. Only one patient showed positive lymph node involvement. No coincidental cases of papillary thyroid carcinoma were present.

Cohen’s Kappa was performed to determine if there was an agreement between radiology and pathology on whether focality was unilateral or bilateral. Cohen’s Kappa was calculated based on patient-level focality (unifocal vs. multifocal). The two modalities agreed on 9 lesions as unifocal and 8 lesions as multifocal. However, the remaining two lesions were diagnosed by radiology as unifocal and as multifocal by pathology. There was good agreement between the two modalities, k = 0.791.

Regarding adjuvant treatment, eleven patients received RAI (nine postoperatively and two for recurrent disease), six patients did not receive it, and the adjuvant therapy data of two patients were not available. During follow-up, seven patients (36.8%) developed locoregional recurrence, while six patients (31.6%) presented with distant metastasis and died later. Of the six metastatic cases, three involved both lung and bone, and one each involved the lung only, bone only, and non-regional axillary lymph nodes. Five of the patients with metastatic disease received RAI, two of them received palliative radiotherapy to the bone, and one received chemotherapy (6 cycles of Gemzar & carboplatin), while the therapeutic data of one patient were unavailable.

**Fig. 3:**
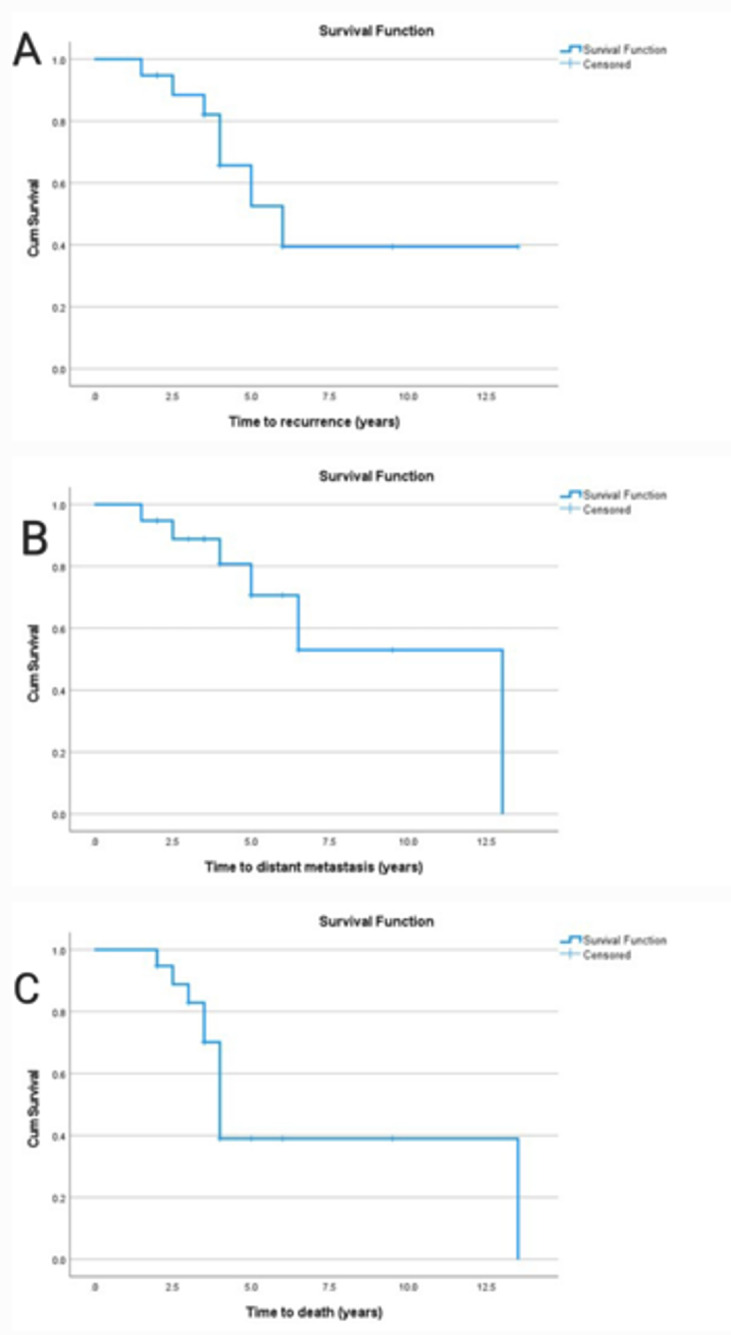
Kaplan-Meier curve for survival distribution of time to recurrence (**A**), distant metastasis (**B**) & death (**C**)

**Fig. 4:**
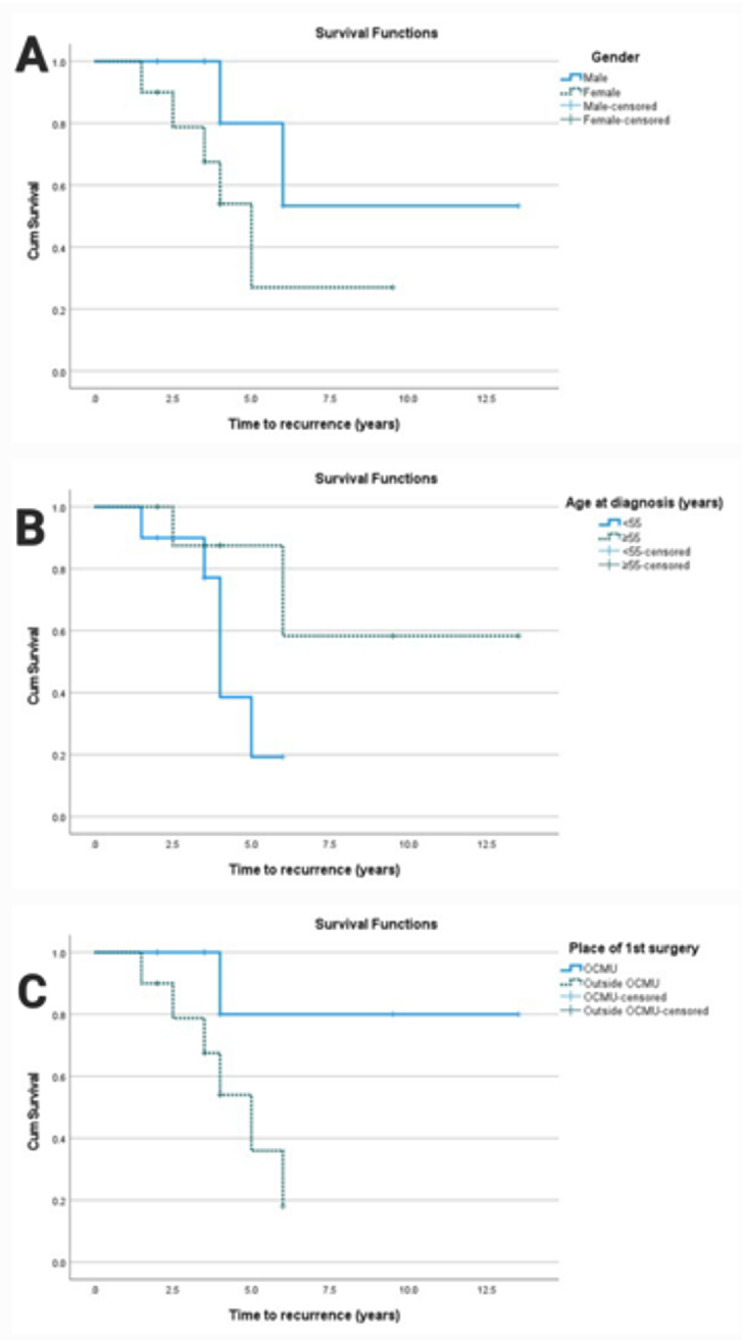
Kaplan-Meier curve for the effect of gender (**A**), age (**B**), and place of first surgery (**C**) on time to recurrence

**Fig. 5:**
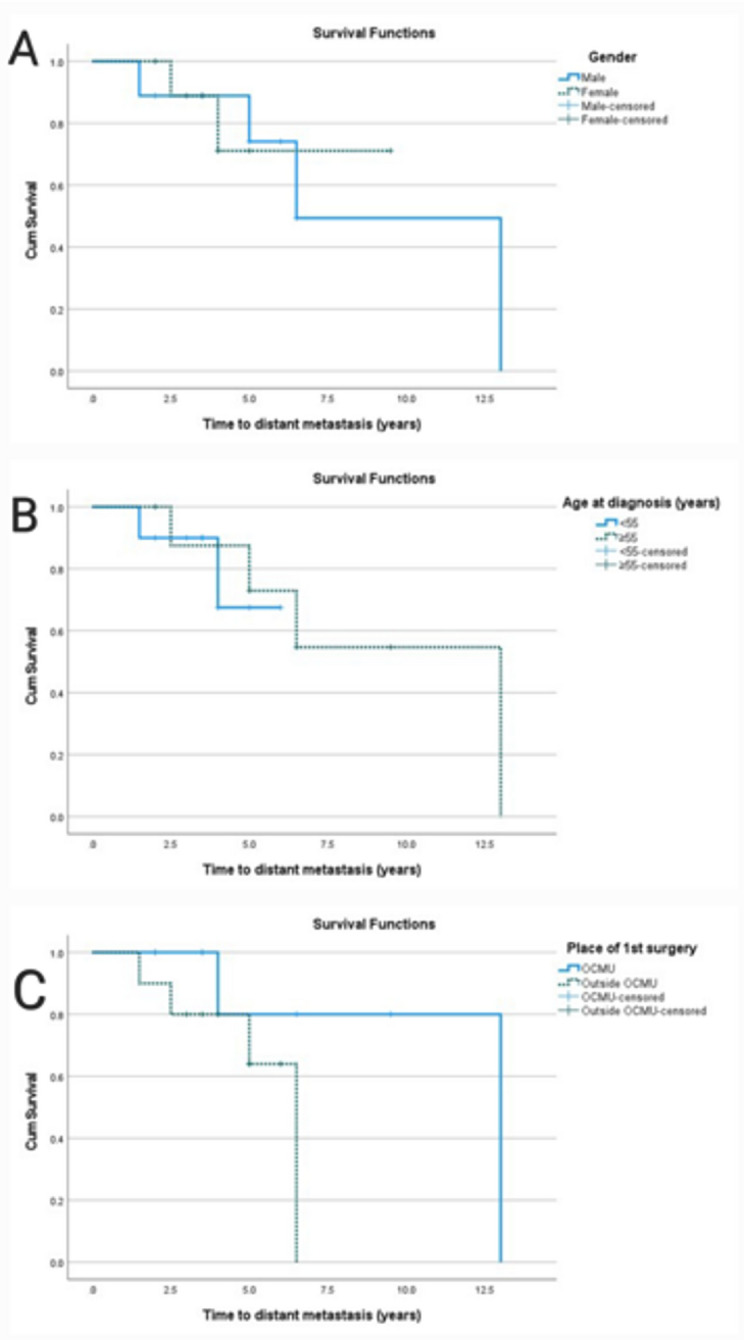
Kaplan-Meier curve for the effect of gender (**A**), age (**B**), and place of first surgery (**C**) on time of distant metastasis

**Fig. 6:**
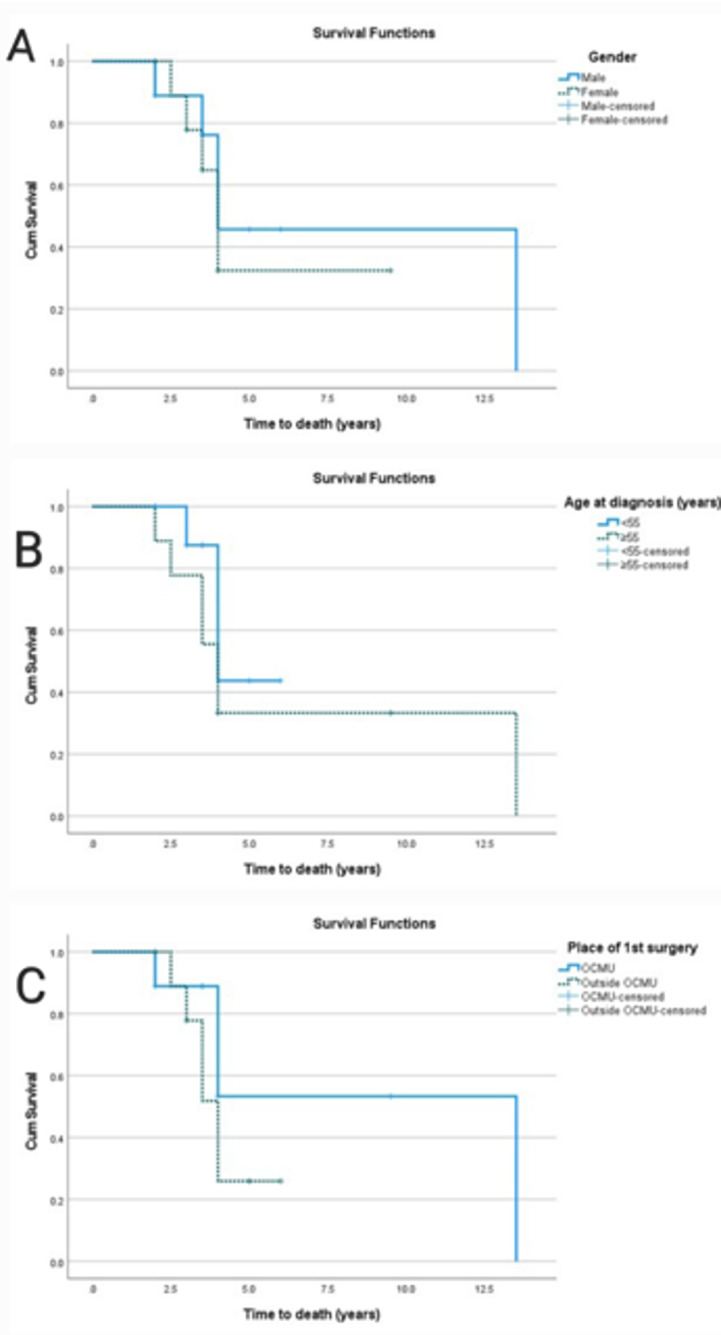
Kaplan-Meier curve for the effect of gender (**A**), age (**B**), and place of first surgery (**C**) on time to death

Using survival analysis (Table 2), the median time to death, distant metastasis, and locoregional recurrence were found to be 4 years (95% CI = 3.6–4.4), 13 years (95% CI = 6.5-NA), and 6 years (95% CI  3.6–8.4), respectively. For locoregional recurrence, the RMST at five years was 4.4 years (95% CI   3.9–4.9), 4.6 years (95% CI  4.1–5.0) for distant metastasis, and 4.1 years (95% CI  3.6–4.5) for overall survival. Along with the impact of age, sex, and surgical setting, Kaplan-Meier survival curves also show how these events are distributed across time (Fig. 3). Risk factors for survival distribution of recurrence, metastasis, and death are presented in Table 3. There was a trend towards worse prognosis in females (Fig. 4), younger patients (Fig. 5), and those who underwent primary surgery outside the center (Fig. 6). These differences did not achieve statistical significance, at least partly due to the small sample size.

**Table 2:** Time to recurrence, metastasis, and death in the studied cases (*n* = 19)

Event	Time to event (years)		RMST at 5 years	
	Median	95% CI	Mean	95% CI
Locoregional recurrence	6	3.6–8.4	4.4	3.9–4.9
Distant metastasis	13	6.5 – NA	4.6	4.1–5.0
Death	4	3.6–4.4	4.1	3.6–4.5

*RMST* Restricted mean survival time. *CI* Confidence interval

**Table 3:** Risk factors for survival distribution of recurrence, metastasis, and death

Risk factor	Time to event		Logrank test		RMST at 5 years		Hazard	
	Median	95% CI	χ^2^ [1]	Sig.	RMST	Sig.	HR	95% CI
Time to locoregional recurrence								
Sex			1.978	*0.160*		*0.090*		
Male	NA	NA – NA			4.8			
Female	5	3.5–6.5			4.1		3.0	0.7–14.1
Age (years)			2.641	*0.104*		*0.179*		
≥55	NA	NA – NA			4.7			
<55	4	3.5–4.5			4.1		3.6	0.8–17
Surgery place			3.255	*0.071*		*0.090*		
Tertiary care center	NA	NA – NA			4.8			
Elsewhere	5	3.3–6.7			4.1		4	0.9–18.2
Time to distant metastasis								
Sex			0.002	*0.967*		*0.886*		
Male	6.5	1.7–11.3			4.6			
Female	NA	NA – NA			4.5		1	0.2–6.1
Age (years)			0.141	*0.707*		*0.574*		
≥55	13	NA – NA			4.7			
<55	NA	NA – NA			4.4		1.5	0.2–11.4
Surgery place			1.907	*0.167*		*0.347*		
Tertiary care center	13	NA – NA			4.8			
Elsewhere	6.5	NA – NA			4.4		3.6	0.6–22.1
Time to death								
Sex			0.252	*0.616*		*0.671*		
Male	4	0–8.9			4.2			
Female	4	3.5–4.6			4		1.5	0.3–6.2
Age (years)			0.872	*0.350*		*0.279*		
≥55	4	3.3–4.7			3.8			
<55	4	3–5			4.3		0.5	0.1–2.1
Surgery place			1.548	*0.213*		*0.294*		
Tertiary care center	13.5	NA – NA			4.3			
Elsewhere	4	3.4–4.6			3.9		2.5	0.6–10.8

*NA* Not available, *RMST* Restricted mean survival time, *Sig* statistical significance (p-value), *HR* Hazard ratio, *CI* Confidence interval, χ^2^ [1] = chi-square at one degree of freedom

## Discussion

Hürthle (oncocytic) cells are large oxyphilic follicular cells characterized by abundant mitochondria—often occupying most of the cytoplasm—and by large nuclei with prominent nucleoli. Mitochondrial DNA alterations may be present, especially in neoplastic lesions [1–3, 13, 14]. In our study, 19 patients were diagnosed with HCC with a slight female predominance (*n* = 10, 52.6%). This differs from the literature, as most published studies report a significant female predominance [15–18]. However, Boonrod et al., reported a similar gender distribution [19]. The ages of our patients ranged from 38 to 77 years, with a median age at diagnosis of 54.8 years, similar to that reported in other studies [15–17, 20]. Other studies reported an older median age [21]. Moreover, there is evidence that HCC can be present in age groups younger than 21 years old [18, 22].

Radiological examination showed that 57.9% of patients had unifocal lesions and 42.1% had multifocal lesions. However, pathological evaluation showed that 52.6% of patients had multifocal lesions. In contrast, the literature reports a much lower percentage for multifocal lesions, ranging from 5% to 28.6% [16, 17, 23, 24]. Most of the cases had a TIRADS score of 4 (*n* = 10, 52.6%), and there is evidence from the literature that most cases are usually classified as TIRAD-4 [20, 25]. However, another study showed that most HCC cases had low suspicion nodules using Korean TIRADS [26].

Pathologically, HCC and follicular thyroid carcinoma are categorized using the same subgroups (minimally invasive, angioinvasive, and widely invasive), but HCC is distinguished from follicular carcinoma by showing invasive malignant follicular cell neoplasms composed of at least 75% oncocytic cells in which the nuclear features of papillary carcinoma -excluding oncocytic PTC and oncocytic encapsulated follicular subtype of PTC- and high-grade features – excluding poorly differentiated oncocytic carcinoma- are absent [2, 3, 27]. The ability of FNAC to differentiate benign from malignant lesions is limited [28], and also to differentiate benign lesions from other mimics as multinodular goiter, Hashimoto thyroiditis [29], and trabecular adenoma [30]. There is a promising role for programmed death-ligand 1 (PD-L1) immunostaining [31] and AI-based tools [32] in improving the sensitivity. In our cohort, Preoperative FNAC classified 68.4% of patients as Bethesda III-IV, in agreement with other studies where HCC cases were classified into Bethesda III to IV [33].

Only one patient had ipsilateral LN (lymph node) involvement. Most of the studies reported a limited number of LN metastases [22, 26, 34]. There were lympho-vascular emboli in 6 patients (31.6%). Comparable results were reported [4, 11, 18]. The pathological median size was 6.7 cm (IQR: 4–8 cm), which is slightly larger in comparison with the literature, which states that the size ranges from 2.5 to 4.8 cm [16, 17, 26].

In our study, most of the patients underwent total thyroidectomy (*n* = 12, 80%) -either at one stage or two stages- and hemithyroidectomy (*n* = 3, 20%). Regarding the three patients who underwent hemithyroidectomy only, two of them had minimally invasive HCC, while the third patient declined completion of surgery. Two patients had a selective lateral neck dissection. Most of the cases in the literature underwent total thyroidectomy and near-total thyroidectomy with neck dissection, either radical or modified. There is evidence also that some cases underwent partial thyroidectomy, hemithyroidectomy, and lobectomy [16, 17, 21, 22, 35, 36]. NCCN guidelines recommend hemithyroidectomy for small tumors with no concerning features, including encapsulated angioinvasive carcinoma with < 4 vessel invasion and minimally invasive carcinoma. Total thyroidectomy is an accepted option for all cases and is a must for invasive cancer (widely invasive or encapsulated angioinvasive with ≥ 4 vessels invasion) [37].

During follow-up, seven patients developed locoregional recurrence (36.8%), while six patients (31.6%) presented with distant metastasis and died later. Three of these metastases were to both the lung and bone, while one was to the lung only, one to bone only, and one to non-regional axillary lymph nodes. This rate of metastasis was higher than reported by other studies. Vogrin et al. reported 14% recurrence in regional lymph nodes [38], while other studies showed that fewer cases (9.4%) were diagnosed with or developed distant metastasis [39]. Two factors may explain this: the small sample size and the nature of the center being a tertiary referral center to which more advanced and complex cases are referred. Systemic treatments, including RAI (*n* = 5), palliative radiotherapy (*n* = 2), and chemotherapy (*n* = 1), were used in metastatic cases, but these treatment protocols showed limited impact on survival, possibly due to late-stage disease, and also the small sample size. RAI is typically recommended for differentiated high-grade carcinoma, gross extrathyroidal extension, extensive vascular invasion, significant N1b disease, bulky or more than five positive LNs, and postoperative unstimulated thyroglobulin > 10 ng/ml. TKIs, such as Lenvatinib and Sorafenib, which are the standard of care in RAI-refractory disease [37], were not administered due to limited availability, high cost, and preference for RAI and palliative therapies in our cohort. This is an important note to help build scientific recommendations based on our local national experience in view of the international guidelines and the local financial capabilities.

Regarding survival analysis, patients needed a median time of 6 (95% CI 3.6–8.4), 13 (95% CI 6.5-NA), and 4 years (95% CI 3.6–4.4) to develop recurrence, distant metastasis, and death, respectively. The RMST was 4.4 (95% CI 3.9–4.9) for LRR, 4.6 (95% CI 4.1–5.0) for distant metastasis, and 4.1 (95% CI 3.6–4.5) for death. Wong et al. reported a median time to recurrence as 2.6 years, and to death as 6.3 years [40]. Using the Kaplan-Meier curve, factors affecting the 5-year probability of recurrence and distant metastasis were female gender, aging less than 55 years, and undergoing surgery outside our center. Factors contributing to the 5-year probability of OS were female gender, aging more than or equal to 55 years, and undergoing surgery outside a tertiary referral center. On the contrary, other studies showed lower 5-year and 10-year probabilities of survival in men and patients aged more than 45 years [2, 20]. Regarding the place of primary surgery, our findings are consistent with the literature documenting that better surgical and oncological outcomes are correlated with higher volume of the treatment centers. Whether defining a high-volume center as performing more than 12 or 27 surgeries for thyroid cancer per year [41, 42], our center exceeds these numbers, as more than 175 thyroid cancer surgeries are performed yearly.

Our study has limitations, including the small sample size due to the low incidence rate of HCC -making the observed trends might be hypothesis-generating rather than confirmatory-, the retrospective design of the study, and the long time period of the study, leading to heterogeneity in surgical and treatment guidelines. Additional limitations include potential selection bias, as patients referred to our tertiary center may have more complex cases, as 4/19 patients presented with recurrent disease, potentially skewing outcomes toward more aggressive behavior, and missing data for a few patients, which may affect outcome analyses. Also, including surgically treated patients only limited the sample size and led to selection bias. However, it presents a fifteen-year yield of a tertiary center experience. Also, the data were prospectively maintained. In addition, adequate statistical analysis was performed.

## Conclusion

Hürthle cell carcinoma is a rare and aggressive thyroid tumor with unique pathological traits and unpredictable behavior. Diagnosing HCC remains challenging due to its overlapping features with other thyroid conditions. Total thyroidectomy with selective neck dissection when indicated remains the preferred therapeutic option. Age, gender, and center volume of the first surgery can affect patient outcomes, particularly recurrence and survival rates.

## Data Availability

All the clinical, radiological, and pathological data used in this manuscript are available on the Mansoura University medical system (Ibn Sina Hospital Management System). [http://srv137.mans.edu.eg/mus/newSystem/]
